# A green marriage: the union of theophylline's catalytic activity and healing potential

**DOI:** 10.1039/d4ra08479a

**Published:** 2025-06-02

**Authors:** Abdul Ahad, Adnan Majeed, Ayesha Zafar, Muhammad Adnan Iqbal, Shahzaib Ali, Muneeba Batool, Asma Rehman, Faiza Manzoor

**Affiliations:** a Department of Chemistry, Government College University Faisalabad Faisalabad 38000 Pakistan; b Department of Chemistry, University of Agriculture Faisalabad Faisalabad 38000 Pakistan adnan.iqbal@uaf.edu.pk; c Organometallic and Coordination Chemistry Laboratory, University of Agriculture Faisalabad Faisalabad 38000 Pakistan

## Abstract

In recent studies, theophylline has been shown to be a green, effective, and biodegradable catalyst suitable for various multicomponent processes. This review explores theophylline's biological synthesis, catabolism, and advanced extraction methods, demonstrating its versatility. Theophylline is utilized to synthesize metal complexes through innovative methods, including the preparation of imidazolium salts and their conversion into N-heterocyclic carbene (NHC) ligands. The therapeutic application of theophylline in the pharmaceutical and medical domains is enhanced by these complexes, which exhibit encouraging potential as antimicrobial and anticancer agents. It exhibits a high yield and efficiency in organic reactions, particularly in acylation, methylation, and nucleophilic substitution reactions, making it a good fit for industrial processes. The review also discusses potential neurological side effects of theophylline and offers prevention and therapy options. It is recommended for future studies to in-depth elucidate theophylline's properties, effects, and uses for improving industrial operations and treating medical conditions. In this thorough analysis, theophylline's adaptability and potential are highlighted, paving the way for more research and development of its wide range of applications.

## Abstract 2

Versatile applications of theophylline: from green catalysis to pharmaceutical and industrial innovations.

## Graphical abstract

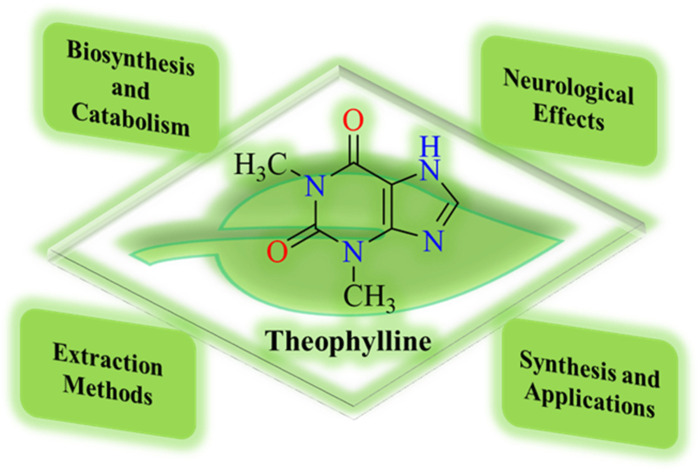

## 1 Introduction

Natural materials are widely available for use in sustainable procedures and drug development.^1–3^ Theobromine, theophylline, and other methylxanthine alkaloids are becoming known as green, bio-renewable catalysts.^4,5^ In particular, theophylline has recently received attention for its potential biological applications.^6^ Cocoa beans and tea contain theophylline, a drug used to treat allergies and chronic bronchitis.^7,8^ It is a member of the xanthine family and was synthesized in 1895 for use as a diuretic.^9,10^ Theophylline was first prescribed as a treatment for asthma in 1937 and is a typical drug taken orally for treating symptoms of asthma and chronic obstructive pulmonary disease (COPD).^11,12^ It has also been used for the treatment of infant apnea and emphysema diseases.^13–15^ Theophylline is currently used as a third-line alternative therapy in developed countries for poorly managed respiratory diseases because inhaled β2-agonists and corticosteroids work better in this regard.^16,17^ Although several theophylline derivatives have been successfully produced, none of them are more effective than the original theophylline.^18,19^ A strong bronchodilator called enprofylline was taken off the market because it was hazardous to the liver.^20^ Theophylline salts such as aminophylline are commonly used to improve solubility for intravenous use.^21^ Because it has a weaker effect on adenosine receptors than doxofylline, it has comparable efficacy but fewer side effects if taken twice daily.^22–24^ Theophylline has been used for more than 70 years, but its exact molecular mechanism and mechanism of action are still unknown.^25^ Several hypothesized mechanisms, such as phosphodiesterase inhibition and adenosine receptor antagonistic effects, as well as impacts on cytokine release and cell death, usually require drug doses higher than those used for conventional therapy.^26^

**Fig. 1:**
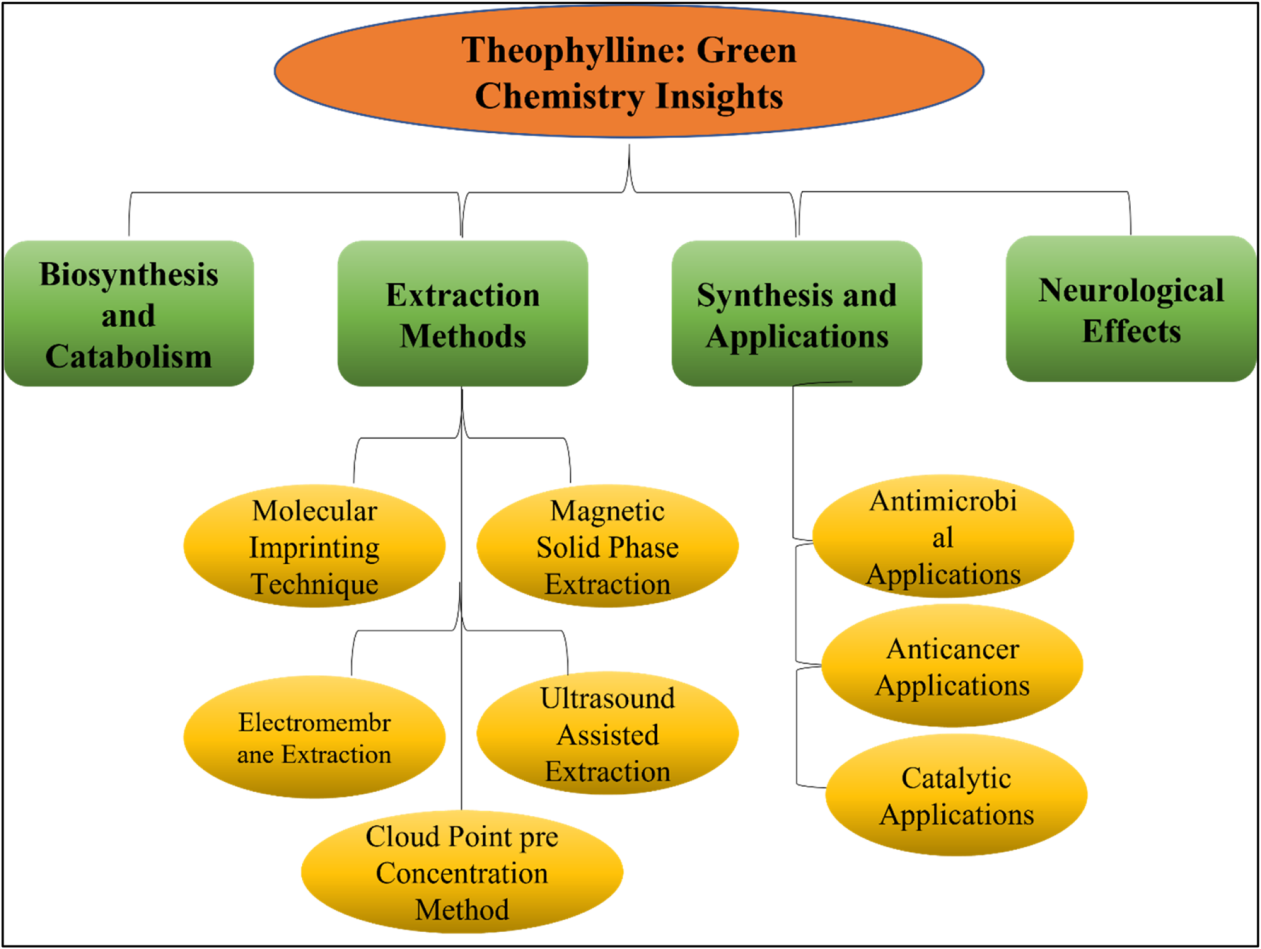
Graphical representation of consolidated review highlights.

Green and sustainable chemistry uses natural and benign resources in organometallic chemistry and catalysis.^27^ These approaches minimize environmental effects, increase efficiency, and reduce dependency on toxic materials, all of which contribute to the development of safer and more sustainable chemical processes.^28–30^ Theophylline is a cheap, widely available chemical that has potential applications as an environmentally friendly, basic catalyst in the synthesis of organic compounds.^31,32^ Its distinct structure, which consists of nitrogen and oxygen atoms, allows it to interact with a wide range of substrates efficiently, possibly stabilizing transition states and chemical intermediates.^33,34^ This has the potential to improve chemical processes' selectivity and efficiency.^35^ By acting as a catalyst, theophylline provides a sustainable solution, eradicating the demand for more expensive or dangerous alternatives.^36^ Due to its availability and low cost, it is a desirable option for researchers looking for environmentally responsible ways to create new organic molecules, which will enhance chemical synthesis in both the short and long term.^37,38^ For the synthesis of metal complexes such as gold, silver, palladium, copper, and platinum, the imidazole ring of theophylline acts as an N-heterocyclic carbene precursor.^39–42^ The chemical structure and reactivity of theophylline make these complexes useful in a variety of coupling reactions, demonstrating the substance's versatility in material science and catalysis. A comprehensive overview of the topic is missing, despite theophylline's potential as a catalyst in a variety of chemical processes. As shown in Fig. 1, this review attempts to bring theophylline's potential as an antibacterial and anticancer agent to light, compile current knowledge on its advanced extraction methods, as well as its application in organic transformations. A variety of examples are provided to highlight developments in this field, and theophylline's function in these processes is demonstrated by describing reaction types, circumstances, yields, and catalytic mechanisms.

### 1.1 Theophylline biosynthesis and catabolism

A xanthine alkaloid called theophylline (1,3-dimethyl-7*H*-purine-2,6-dione) is present in dried mate, coffee, chocolate, and black tea.^43,44^ When taken orally, it absorbs quickly and reaches peak serum levels in 1.5–2 hours.^44^ With a serum half-life ranging from 3 to 12.8 hours, theophylline diffuses into fat-free tissues and undergoes significant hepatic metabolization by cytochrome P450 enzymes. Doses ≥ 7.5 mg kg^−1^ may cause toxicity.^45^ Heart failure, liver, or lung disorders can all affect its elimination, but smokers and children under 16 may have higher removal. Drug interactions cause higher metabolism with phenytoin and rifampicin and lower clearance with erythromycin.^6,46^ Theophylline targets particular kinases essential for retroviral DNA integration, which can prevent HIV-1 replication.^47^ When administered at therapeutic doses, theophylline increases histone deacetylase activity, which affects how inflammatory genes are transcribed in macrophages and epithelial cells.^48^

**Fig. 2:**
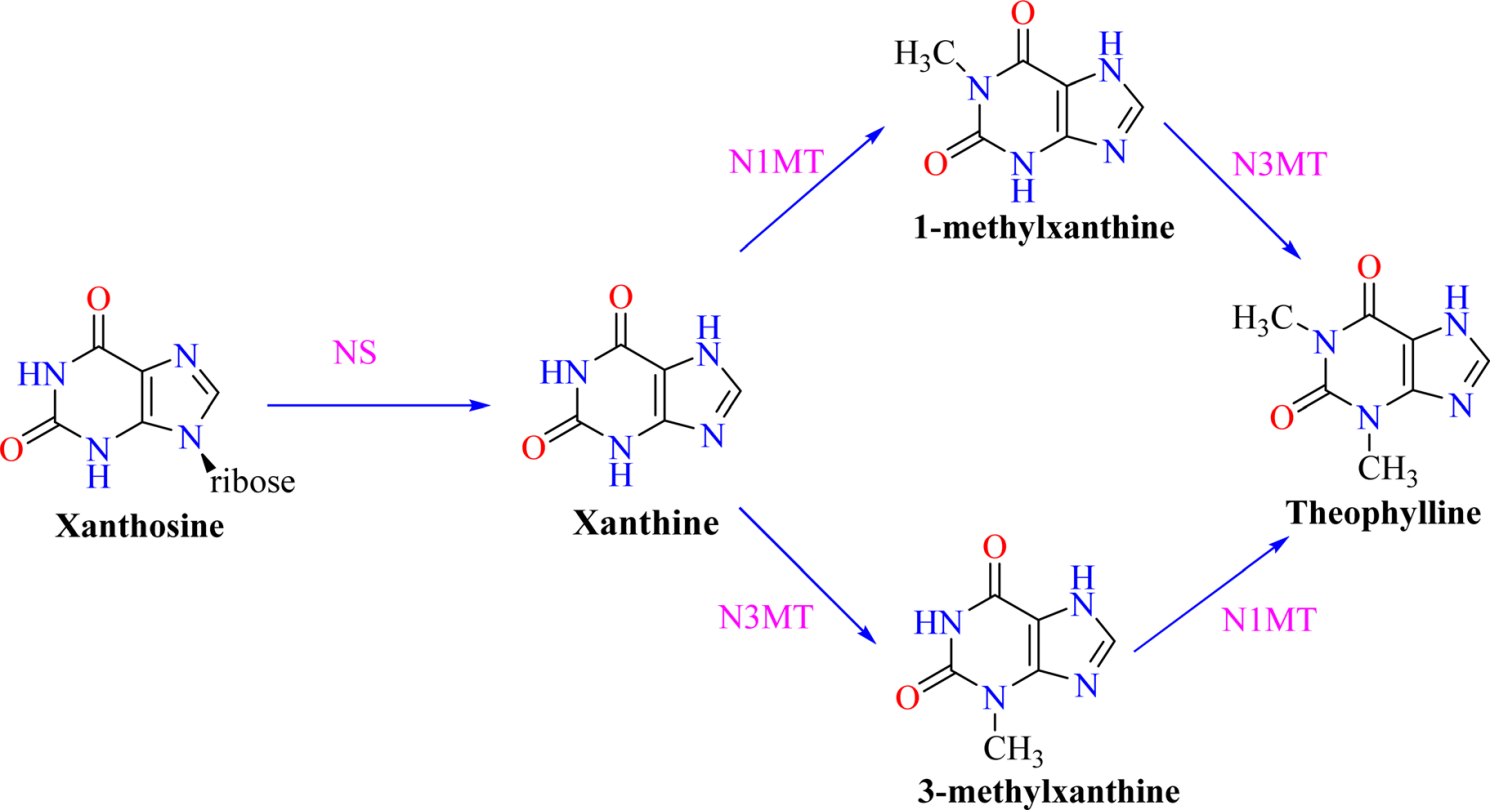
Optimized strategy for *de novo* theophylline synthesis. Enzymes are abbreviated as NS, nucleosidase; N1MT, N_1_-methyltransferase; and N3MT, N_3_-methyltransferase.

**Fig. 3:**
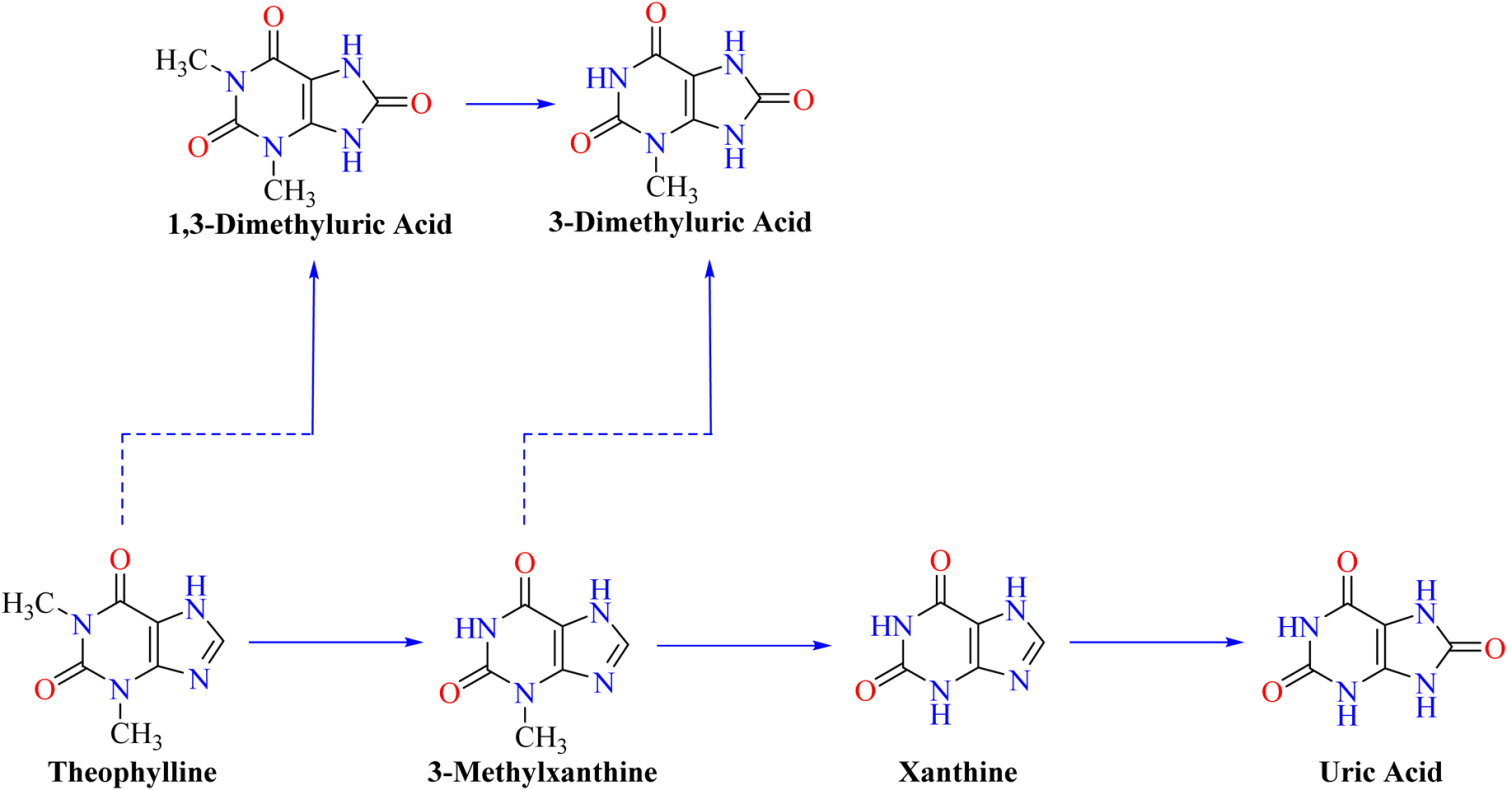
Hypothetical catabolic pathways for theophylline.^53^

Notwithstanding these results, more research is necessary to fully understand the particular molecular pathways driving these impacts.^49^ Theophylline can be prepared synthetically by processes such as methylating xanthines, or it can be extracted from natural sources.^50,51^ Although there are several potential biosynthetic routes from plants to theophylline, the main one entails changing xanthosine into theophylline and is represented in Fig. 2. Herein, xanthosine was initially catalyzed by nucleosidase (NS) to produce xanthine. The process of theophylline biosynthesis involves methylating xanthine at the N1 position to produce 1-methylxanthine, which is then further methylated at the N3 position to produce theophylline, which is an essential component of the pathway.^52^Fig. 3 depicts the catabolic pathways for converting theophylline into smaller units such as 1,3-dimethyluric acid, 3-methylxanthine, xanthine, and uric acid.^53^

### 1.2 Theophylline extraction methods

Conventional theophylline extraction methods from natural sources include ion mobility spectrometry,^54^ reversed-phase high-performance liquid chromatography,^55^ sol–gel process,^56^ maceration,^57^ high-performance liquid chromatography/mass spectrometry (HPLC/MS),^58^ solid phase extraction,^59^ fluorescent assays,^60^ colorimetric assays,^61^ surface plasmon resonance,^62^ and reflux extraction.^63^ Colorimetric and fluorescent assays provide quick and accurate quantification, whereas HPLC/MS is utilized to separate and identify theophylline in complicated extracts. During the extraction process, theophylline's interactions can be detected using surface plasmon resonance, which improves efficiency and purity evaluation. Currently, there are more eco-friendly extraction techniques available than ever before, as illustrated in Table 1, providing an alternative to conventional methods that are less harmful to the environment.

**Table 1:** Comparative extraction methods for isolating theophylline from natural sources^a^

Techniques	Key features	References
Electro-membrane extraction (EME)	EF = 42%, ER = 24%, pH sample solution = 9, pH accepter solution = 13, voltage = 50 V, time = 10 min, RSD = <7%, SLM organic solvents	64
	This technique was developed to improve the extraction process's selectivity, speed, and concentration from aqueous samples	
Molecular imprinting technique (MIT)	The functional monomer was MAA (2.0 mM), EGDMA (1.60 mL), and a 24 hour cross-linking period was ideal. Adsorption capacity = 14.55 mg g^−1^, imprinting factor = 1.26, thermal stability < 220 °C	65
	This method used selective enrichment of THP for pretreatment to identify harmful wastewater	
Ultrasonic-assisted micelle-mediated extraction and cloud point pre-concentration method	TP was extracted from green tea leaves in these methods using non-ionic surfactants Triton X-114 and Genapol X-080. Compared with the factorial design, the uniform design approach requires fewer experiments, which ensures accurate results. The method's precision, accuracy, recovery, calibration, and specificity were all validated	66
Ultrasound-assisted surfactant-enhanced emulsification microextraction	In this eco-friendly method, surfactants and ultrasonic radiation improve solvent dispersion and make emulsification easier	67
	Decanoic acid was the most efficient surfactant, and chloroform was the ideal solvent. For cloudy solution production, the optimal conditions were pH 5.5, acetate buffer, and 3 min of ultrasound treatment. The temperature had no obvious impact on the analytical signal; however, NaCl modestly decreased extraction efficiency	
Magnetic solid phase extraction (MSPE)	The ideal extraction parameters were pH 6, 30 °C, 1.0 mg of adsorbent, pre-ultrasound treatment, and 20 min of adsorption. 3 mL of 50 mM Na_3_PO_4_ (pH 12) and a 5 minute desorption period were employed for the desorption process	68
	Analytes are quickly and selectively concentrated using a magnetic field through MSPE, which combines with HPLC to provide high TP sensitivity. Despite being effective and completed in 30 min, its poor recovery requires further optimization	
High-performance liquid chromatography with diode array detection (HPLC-DAD)	The procedure used a Purospher STAR RP-8 column in isocratic mode with a water–THF–acetonitrile mobile phase at pH 8 and 0.1 M NaOH. Analytes at 273 nm were found and quantified using standard calibration curves under the following conditions: 0.8 mL min^−1^ flow rate, 25 °C column temperature, and 5 min run time	69
Ultrasound-assisted extraction	The fixed parameters for the instrument were 37 kHz, 50% power, 35 °C, and 10 min. A Doehlert matrix was then used to maximize the target compound's extraction from a 30 mg sample	70
	Quick and simultaneous chemical extraction with a low volume of acetone was possible because of ultrasonic energy	

a EF (enrichment factor), ER (extraction recovery), RSD (relative standard deviation), SLM (supported liquid membrane), MAA (methacrylic acid), EGDMA (glycol dimethacrylate), TP (theophylline).

## 2 Synthesis and applications

### 2.1 Theophylline-based antimicrobial complexes

**Scheme 1:**
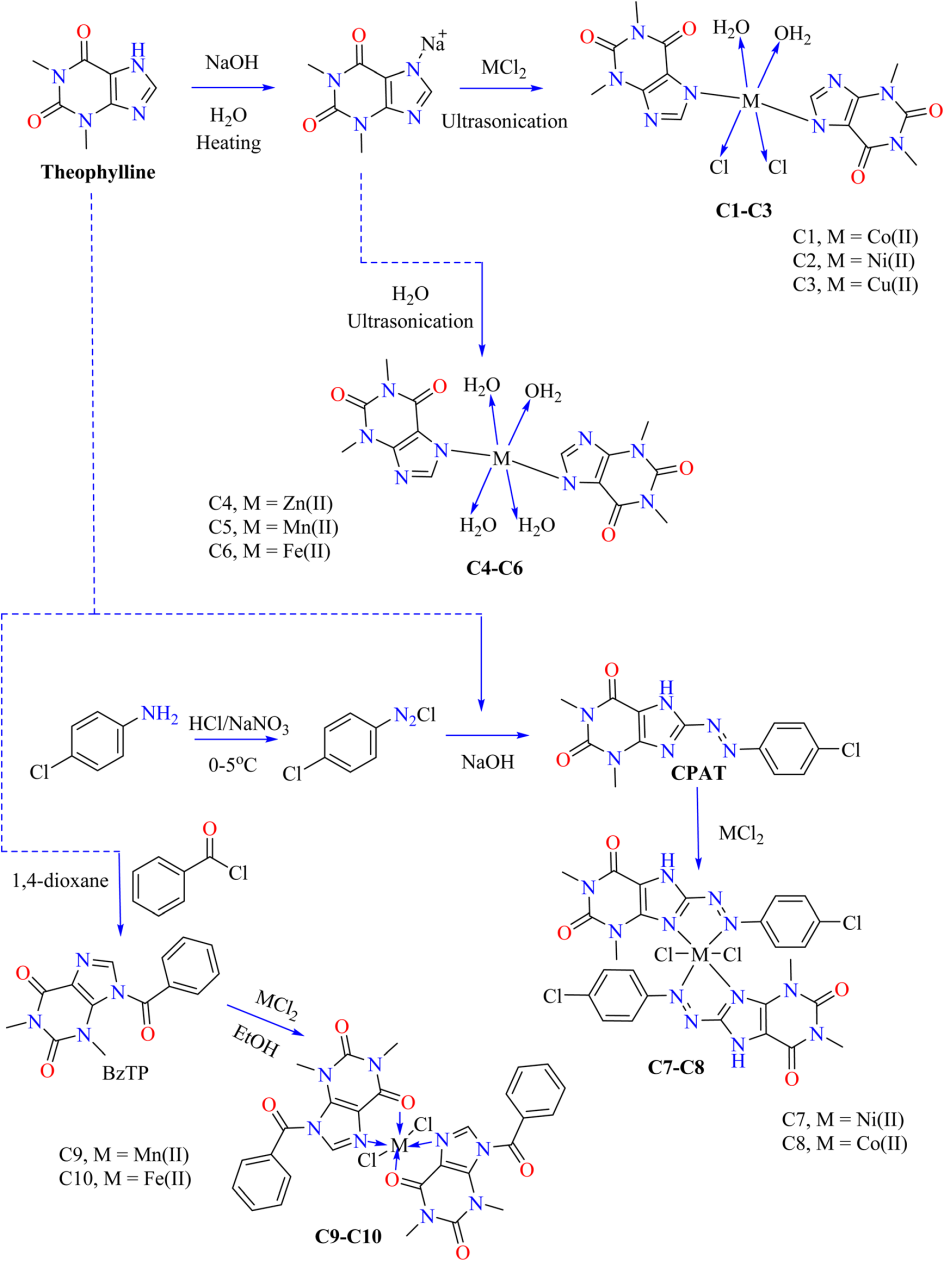
Synthetic route for the synthesis of theophylline-based metal complexes C1–C10.

Scheme 1 shows the optimized synthetic pathways for the synthesis of theophylline-based metallic complexes (C1–C10). Theophylline was dissolved in NaOH, heated, and combined with metal chlorides to produce three nanocomplexes (C1–C3), respectively containing Co(ii), Ni(ii), and Cu(ii) ions. The mixture was sonicated for three hours at 70 °C, allowed to crystallize at room temperature, filtered, cleaned, and allowed to dry in the air as shown in Scheme 1. The nanocomplexes showed 98–100% inhibition zones and significant antibacterial activity against Gram-positive and Gram-negative bacteria (Table 2).^71^ By successfully rupturing bacterial cell membranes and preventing growth, copper nanocomplex C3 attained 100% inhibition.^72^ Ismail and his coworkers dissolved theophylline in 30 mL of NaOH and heated it to 70 °C to produce three nanoscale therapeutic complexes (C4–C6). After adding metal(ii) chloride, the mixture was subjected to three hours of sonication at 70 °C. Crystals developed overnight at room temperature. After that, they were filtered, cleaned with a hot water wash, and allowed to air dry. The antibacterial activity of the complexes was tested against various bacterial strains, as their inhibition zone in Table 1, According to this, the newly synthesized complexes (C4–C6) have excellent potential to kill bacteria.^73^ The process for producing the azo dye of theophylline (CPAT) involved diazotizing 4-chloroaniline, which then reacted with theophylline in a basic solution to produce a dark yellow azo dye. Then, coordination compounds of nickel (C7) and cobalt (C8) were synthesized by reacting the metal salts in hot ethanol with CPAT dye. After the complexes (C7–C8) were filtered, 75% of the yellowish-green C7 complex and 80% of the brown C8 complex were obtained. *Escherichia coli* and *Staphylococcus aureus* were used to test the antibacterial activity of CPAT dye and its metal complexes (C7–C8). Complex C7 exhibited higher antibacterial activity than the dye alone and conventional drug ciprofloxacin (30 mm against *E. coli* and 25 mm against *S. aureus*). Chelation is thought to have increased membrane permeability and lipophilicity, leading to enhanced activity.^74^ After interacting theophylline with 0.01 mol of benzoyl chloride in dioxane for five hours, cooling, and hot water washing, a theophylline-based novel BzTP ligand was formed. Mn(ii) (C9) and Fe(ii) (C10) nanocomplexes were produced by dissolving 0.002 mol of BzTP in ethanol, adding metal chlorides in a 1 : 2 ratio, and then heating the mixture to 50 °C using ultrasonic reflux. After the crystals grew overnight, they were air-dried and cleaned with ethanol. The antibacterial results based on the inhibition zone, as shown in Table 2, indicated that the metal complexes C9–C10 were more potent than the free ligand BzTP. Meanwhile, complex C9 has a higher antibacterial potential against bacterial strains compared with complex C10 and free ligand.^75^

**Table 2:** The inhibitory zone illustrates the impact of theophylline-based metallic complexes on several bacterial species^a^

Compounds	Gram-positive strains		Gram-negative strains	
	*Staphylococcus aureus*	*Bacillus subtilis*	*Klebsiella pneumonia*	*Escherichia coli*
C1	42 mm	Out scale	46 mm	49 mm
C2	37 mm	—	42 mm	35 mm
C3	49 mm	—	Out scale	Out scale
C4	Out scale	39 mm	—	40 mm
C5	—	36 mm	45 mm	40 mm
C6	—	41 mm	49 mm	43 mm
CPAT	10 mm			35 mm
C7	25 mm			30 mm
C8	20 mm			25 mm
Ciprofloxacin	25 mm			15 mm
C9	4 mm	12 mm	8 mm	5 mm
C10	2 mm	9 mm	3 mm	4 mm
BzTP	Out scale	5 mm	2 mm	Out scale
C11	0.03 mm			0.02 mm
CTP	89.67 mm			84.52 mm
Ampicillin	Out of scale			Out of scale
TP	12 mm	6 mm		7 mm
C12	12 mm	12 mm		11 mm
C13	19 mm	16 mm		16 mm
C14	12 mm	6 mm		7 mm

a Out scale = outside the limits of the dish.

**Scheme 2:**
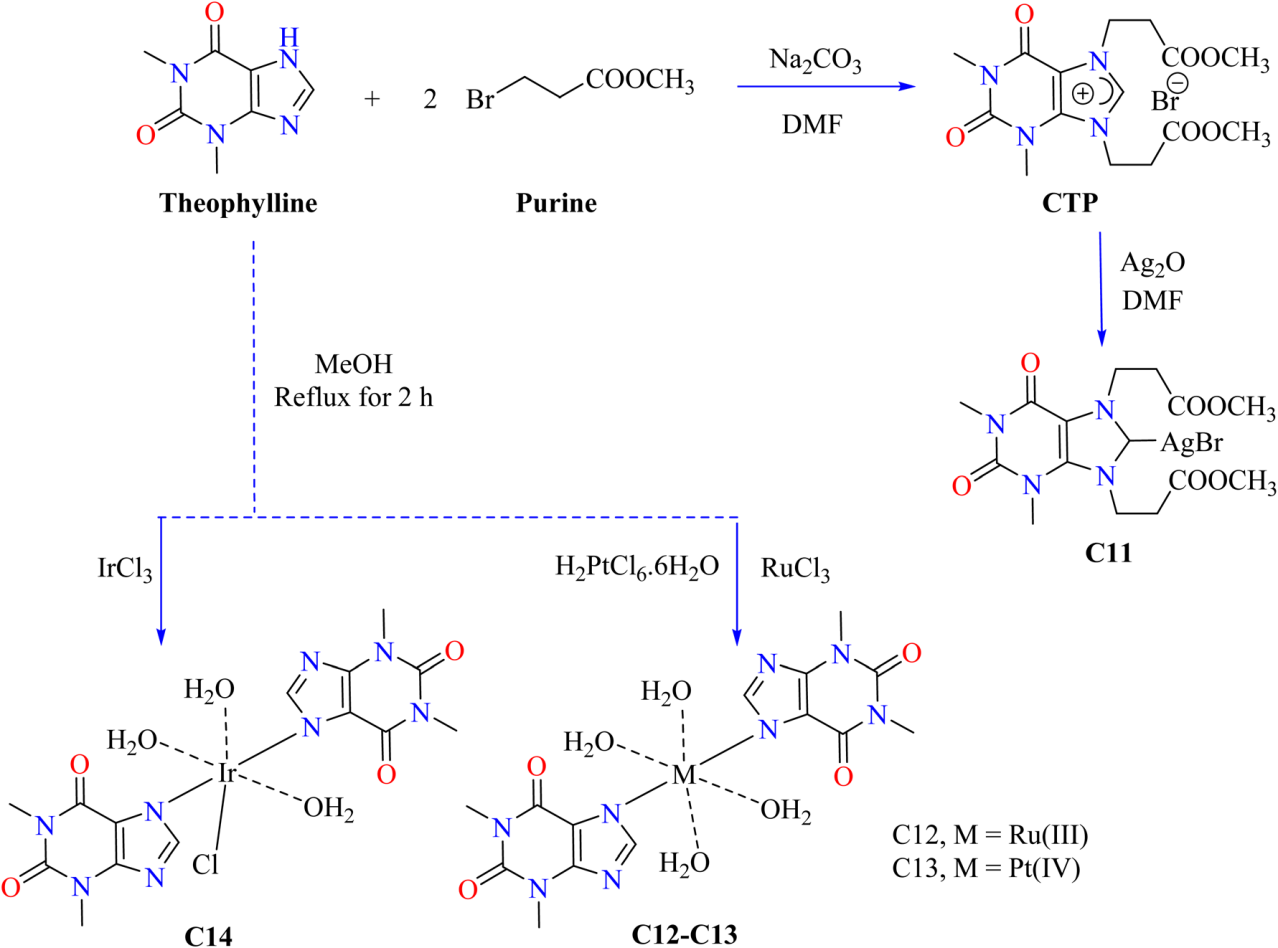
Methodology for the synthesis of theophylline complexes C11–C14.

Carbenes are divalent carbon species that are reactive and possess lone pairs, which help to form new carbon–carbon bonds during chemical processes.^76,77^ M. Donmez *et al.* heated purine, theophylline, and Na_2_CO_3_ to 153 °C for 14 hours at room temperature and then treated it with DMF under argon (Scheme 2). The brown mixture of carbene containing theophylline compound (CTP) was extracted using water and dichloromethane, and theophylline was subsequently obtained by purifying it using column chromatography. After that, CTP and Ag_2_O were reacted in DMF, then filtered while the solvent was evaporated. CH_2_Cl_2_–Et_2_O crystallized to produce the resultant white complex of silver (C11). Ampicillin was utilized to evaluate the antibacterial activity of the combination C11 and its ligand CTP. The findings indicated that NHC carbene ligand CTP is less effective than the silver(i) complex C11 in terms of antibacterial activity.^78^ Metal ions such as ruthenium(iii), platinum(iv), and iridium(iii) were used to synthesize theophylline complexes C12–C14 by Abeer A. El-Habeeb and associates. The metal ions used the deprotonated NH group at nitrogen N7 to form mono-dentate chelates with TP. The synthesis involved mixing metal salts (RuCl_3_, H_2_PtCl_6_·6H_2_O, and IrCl_3_·*x*H_2_O) with 2.0 mmol of TP in MeOH, refluxing for 2 h, and neutralizing with NH_4_OH to achieve pH 8 as shown in Scheme 2.

The antimicrobial activity of C12–C14 complexes was evaluated *in vitro* using both Gram-positive and Gram-negative bacterial strains. The results indicated that the C13 complex has a more comprehensive antibacterial efficacy than free TP and the other complexes. However, the C12 and C14 complexes show enhanced inhibition of *Bacillus subtilis* and *Escherichia coli* compared with free TP. On the other hand, the C13 complex showed a broader spectrum of antibacterial activity. It effectively inhibits Gram-positive bacteria, such as *Bacillus subtilis* and *Staphylococcus aureus*, as well as Gram-negative bacteria, like *Escherichia coli*.^79^ The complex becomes more lipophilic and the electron delocalization owing to chelation reduces the polarity of the metal ion and facilitates the disruption of microbial enzyme binding sites and lipid membrane penetration.^80,81^ However, metal salts alone are less potent as antibacterial agents despite having a higher activity because of their toxicity and potential to bind to biological ligands.^82^

**Fig. 4:**
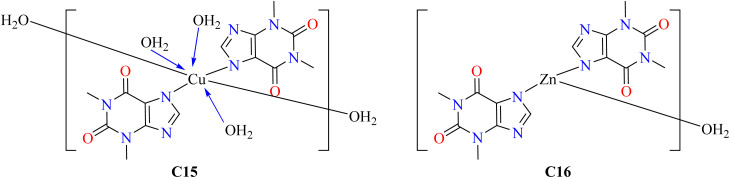
Structural representation of theophylline-based complexes C15 and C16.

Gacki *et al.*^83^ synthesized Cu(ii) and Zn(ii) based theophylline complexes C15 and C16 respectively, as shown in Fig. 4. After the synthesis of complexes, gradual evaporation of an aqueous/ethanol solution produced single crystals suitable for X-ray examination. Two weeks later, the crystals formed, and they remained stable at room temperature. In polar organic solvents such as methanol, ethanol, DMF, and DMSO, these compounds dissolve effectively, while they are only moderately soluble in water. Compared with complex C15 and pure theophylline, complex C16 exhibits a greater capacity for radical scavenging. Theophylline alone is inactive against both Gram-positive and Gram-negative species, although both complexes (C15–C16) have moderate to mild action against Gram-positive rods but are inactive against Gram-negative rods. It will take further research to fully understand its effect.^83^

### 2.2 Theophylline-based anticancer complexes

**Fig. 5:**
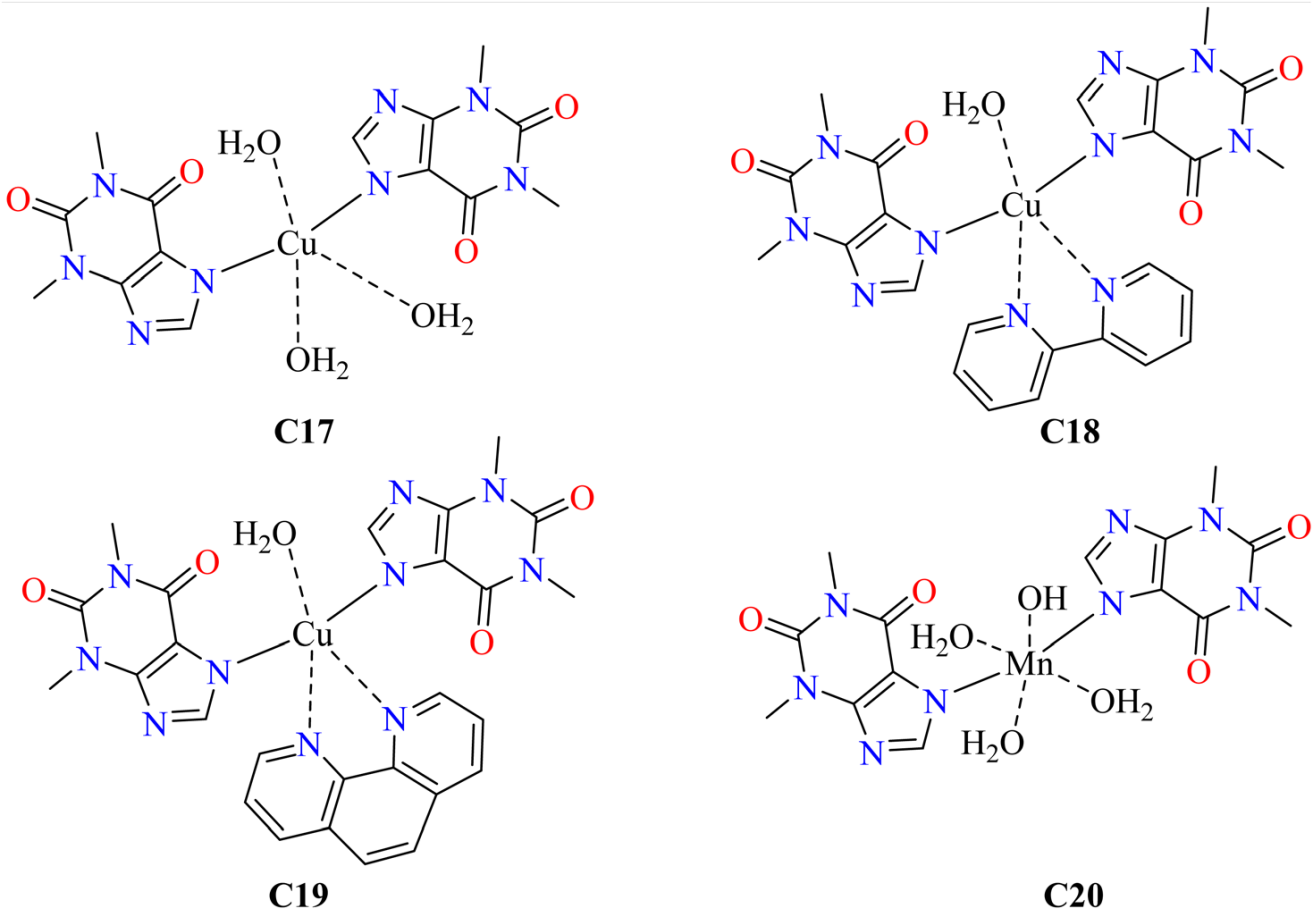
Structural representation of theophylline-based anticancer agents.

Theophylline provides a variety of coordination chemistry due to its N and O donor atoms. In strong circumstances, it can coordinate with metals through N(7)–O(6), or it can behave as a bidentate ligand through N(9) when protonated at pH < 5.^42,84,85^ It coordinates *via* N(7) with metals under neutral to basic conditions. Complexes of copper and manganese with different ligands have demonstrated encouraging anticancer properties.^86–88^ Manganese has been shown to enhance cellular activities and inhibit prostate cancer cell development, while copper complexes have shown cytotoxicity *in vitro*.^89–92^ To investigate the possible anticancer characteristics of 1,2-bipyridine and 1,10-phenanthroline as auxiliary ligands, a researcher focused on manufacturing and assessing Cu(ii) and Mn(ii) theophylline complexes. Theophylline complexes (C17–C20) were prepared by dissolving the theophylline drug using sodium hydroxide and mixing it with metal nitrate solutions. The complexes were then stirred for 4 h at room temperature and high-quality crystals of the resulting complexes were observed 4 weeks later.^93^ Numerous cancer cell lines were used to evaluate complexes C17–C20 (Fig. 5). Meanwhile, complexes C17 and C20 failed to demonstrate concentration-dependent effects, with complexes C18 and C19 reducing growth in a dose-dependent way. Complex C18's clinical usefulness was limited due to its IC_50_ values exceeding 50 μM, which is not within a clinically significant range.^94^ All studied cancer cell lines showed strong activity in response to complex C19, even severe ones like glioblastoma, pancreatic cancer, and triple-negative breast cancer (Table 3). Its IC_50_ value varied from 1.5 μM to 4.9 μM, with the most sensitive cells being MDA-MB231. This selectivity is encouraging, even though it was less effective against normal MCF-10A cells. Since doxorubicin has a proven track record of clinical success, it served as a positive control.^95^ Animal models should be used to further assess adverse effects.

**Table 3:** Half-maximal inhibitory concentration (IC_50_) of theophylline-based anticancer complexes

Compounds	Cell line	Cancer type	IC_50_ value (μM)	Doxorubicin IC_50_ value (μM)
C18	SH-SY5Y	Neuroblastoma	1.74 ± 0.26	1.49 ± 0.13
	MIA PaCa-2	Pancreatic	2.44 ± 0.13	14.31 ± 0.36
	SF268	Glioblastoma	3.17 ± 0.17	1.82 ± 0.25
	A549	Lung	4.9 ± 0.37	7.12 ± 0.34
	MDA-MB-231	Breast	1.5 ± 0.05	9.45 ± 0.14
	MCF7	Breast	2.63 ± 0.18	2.26 ± 0.14
	HT-29	Colorectal	3.1 ± 0.26	51.67 ± 2.02
	MCF-10A	Breast (normal)	7.6 ± 0.25	3.8 ± 0.52
C21	PC-3	Prostate	7.8 ± 0.4	—
	SKLU-1	Lung	10.7 ± 0.7	—
C30g	A549	Lung	1.25 ± 1.6	—

**Scheme 3:**
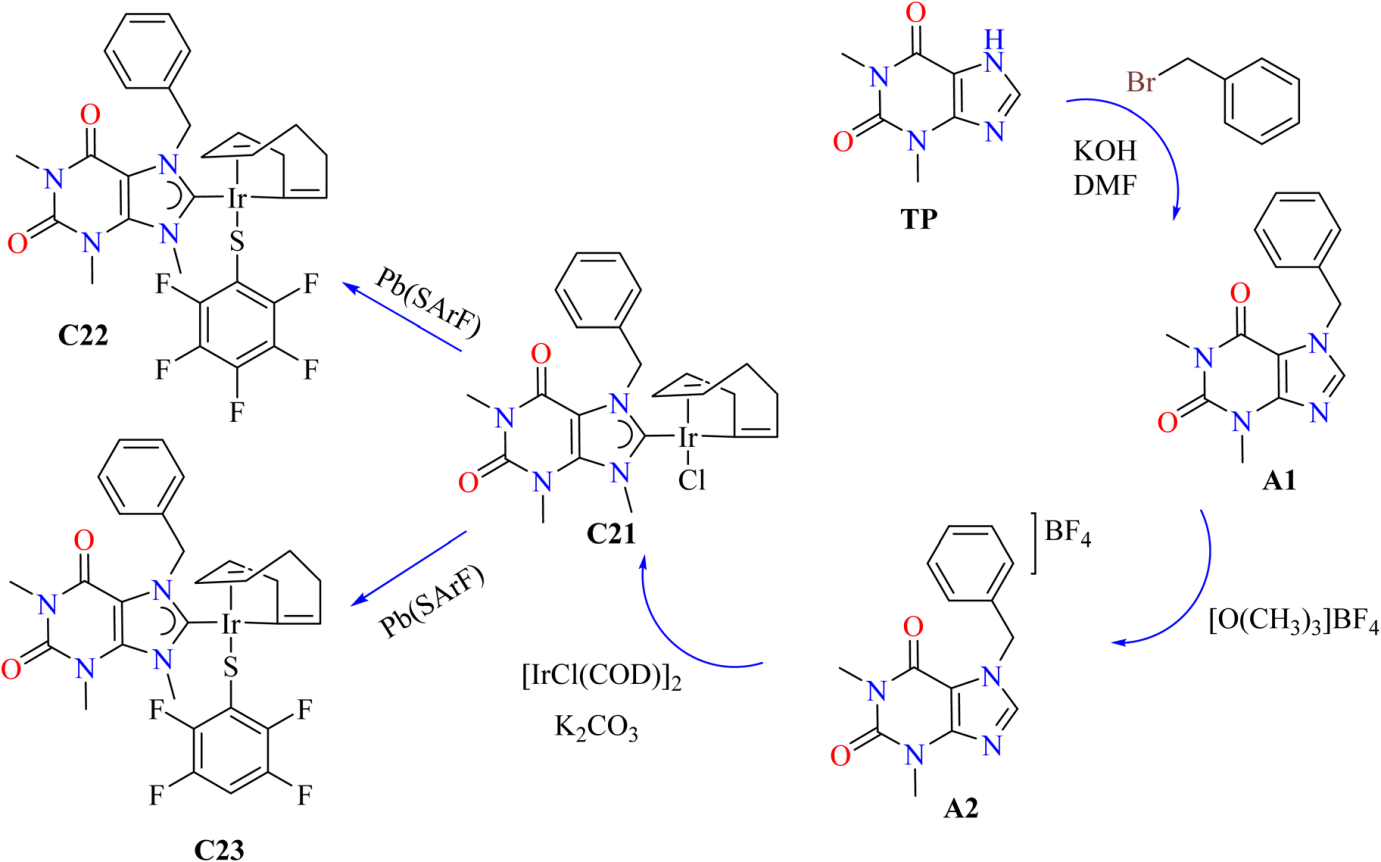
Synthesis of theophylline-based N-heterocyclic iridium complexes C21–C23.

Iridium, particularly in the +3 oxidation state, can act as antiproliferative agents, such as Ir(iii) complexes.^96–98^ Theophylline was reacted with KOH and benzyl bromide to produce 7-benzyl-1,3,9-trimethylxanthinium tetrafluoroborate (A2), which was then treated with [O(CH_3_)_3_]BF_4_. After that, this compound and iridium were combined to synthesize complex (C21) with a 41% yield (Scheme 3). Good yields of the required complexes were obtained by substituting aromatic fluorinated thiolates for the chloride ligand using [Pb(SArF)_2_] in acetone. Theophylline derivatives (A1) and (A2) were shown to be mostly inert against six human cancer cell lines in initial *in vitro* cytotoxicity studies employing 25 μM compounds in DMSO. On the other hand, Ir(i) complexes C21–C23 showed significant activity. Because of their advantageous non-covalent interactions with fluorinated aromatic rings, thiolate derivatives C22 and C23 demonstrated above 98% inhibition in all examined lines. As indicated in Table 3, complex C21 outperformed cisplatin in PC-3 but was less effective in SKLU-1, exhibiting selective action with IC_50_ values of 7.8 ± 0.4 μM for PC-3 and 10.7 ± 0.7 μM for SKLU-1, as shown in Table 3.^99^

**Scheme 4:**
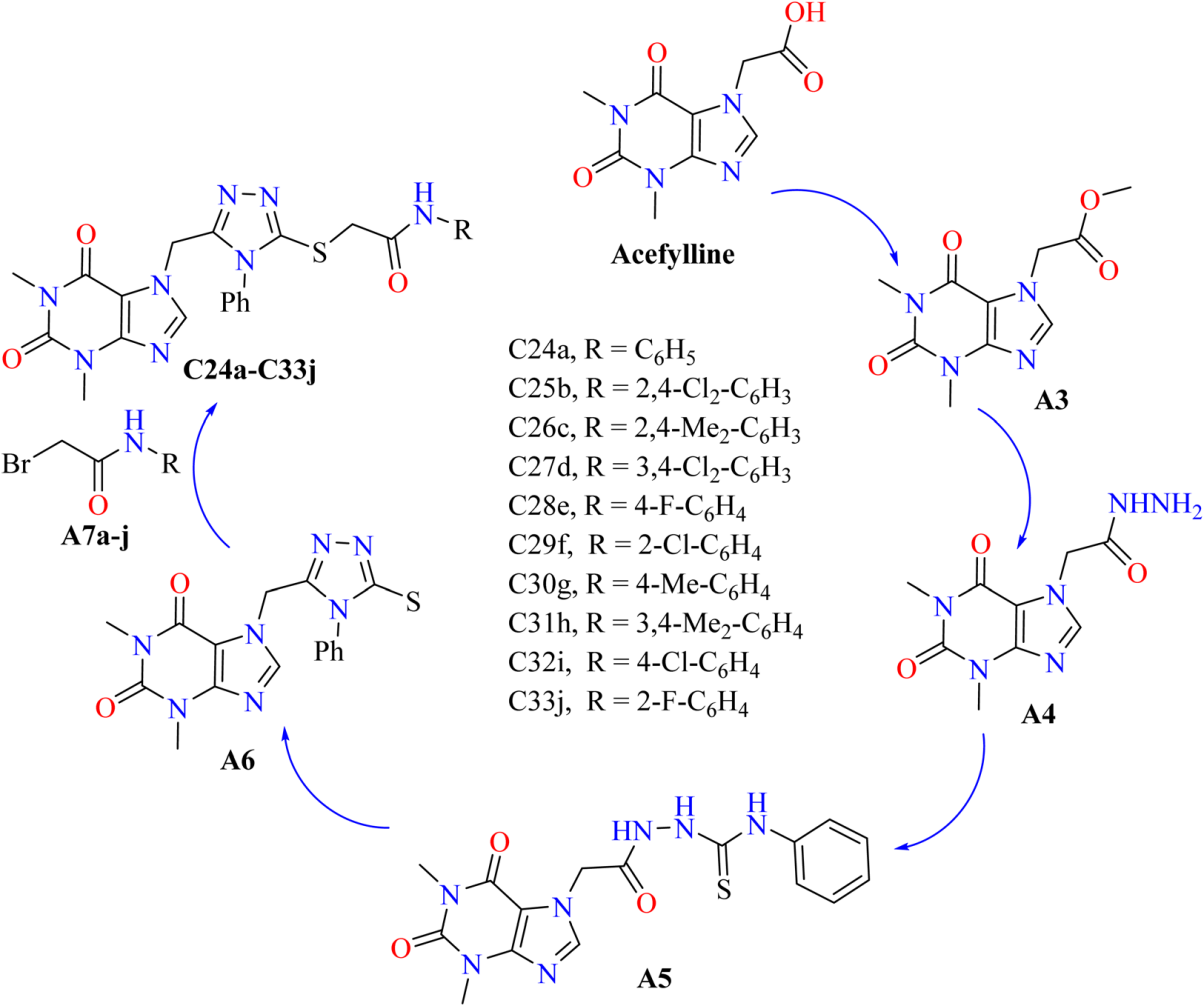
Reaction pathway for the synthesis of theophylline-based anticancer compounds.

Acefylline, an active theophylline derivative, is utilized as a smooth muscle relaxant, bronchodilator, diuretic, and heart stimulant.^100^ Apart from its known applications, its amides and methyl ester groups show noteworthy efficacy against myeloid leukemia cells, *Mycobacterium tuberculosis*, and many cancer cell lines.^101–103^ Its wide range of activities highlight its promise as a therapeutic agent and serve as a foundation for the development of novel treatments for transmissible diseases and cancer. The synthesis of acefylline derivatives involve multiple stages. As illustrated in Scheme 4, theophylline-7-acetate (A3) was initially produced by esterifying acefylline with methanol and sulfuric acid. Theophylline-7-acetohydrazide (A4) was then produced by reacting A3 with hydrazine monohydrate.^103^ After phenyl isothiocyanate treatment of intermediate B, thiosemicarbazide (A5) was produced. This compound was then hydrolyzed to produce the acefylline–triazole hybrid (A6).^104^ Following a reaction between bromoacetyl bromide and a variety of aromatic amines, 2-bromo-*N*-substituted phenyl acetamides (A7a–j) were formed.^105^ These were subsequently coupled with A6 in dichloromethane for the production of target compounds (C24a–C33j). All of the compounds examined showed significant inhibitory potential when their IC_50_ values were evaluated against the lung cancer cell line A549. With an IC_50_ value of 1.25 ± 1.6 μM, compound C30g and acefylline were shown to be the most effective anti-cancer derivative among them. There was a mild cytotoxic activity shown by compounds C28e and C32i, with cell viabilities of 54.82 ± 4.88% and 50.82 ± 2.78%, respectively. On the other hand, compounds C25b, C29f, and C33j showed greater values for cell survival, indicating a lower level of anti-cancer action and placing them as the least effective in this investigation.^106^

### 2.3 Role of theophylline as a catalyst

**Scheme 5:**
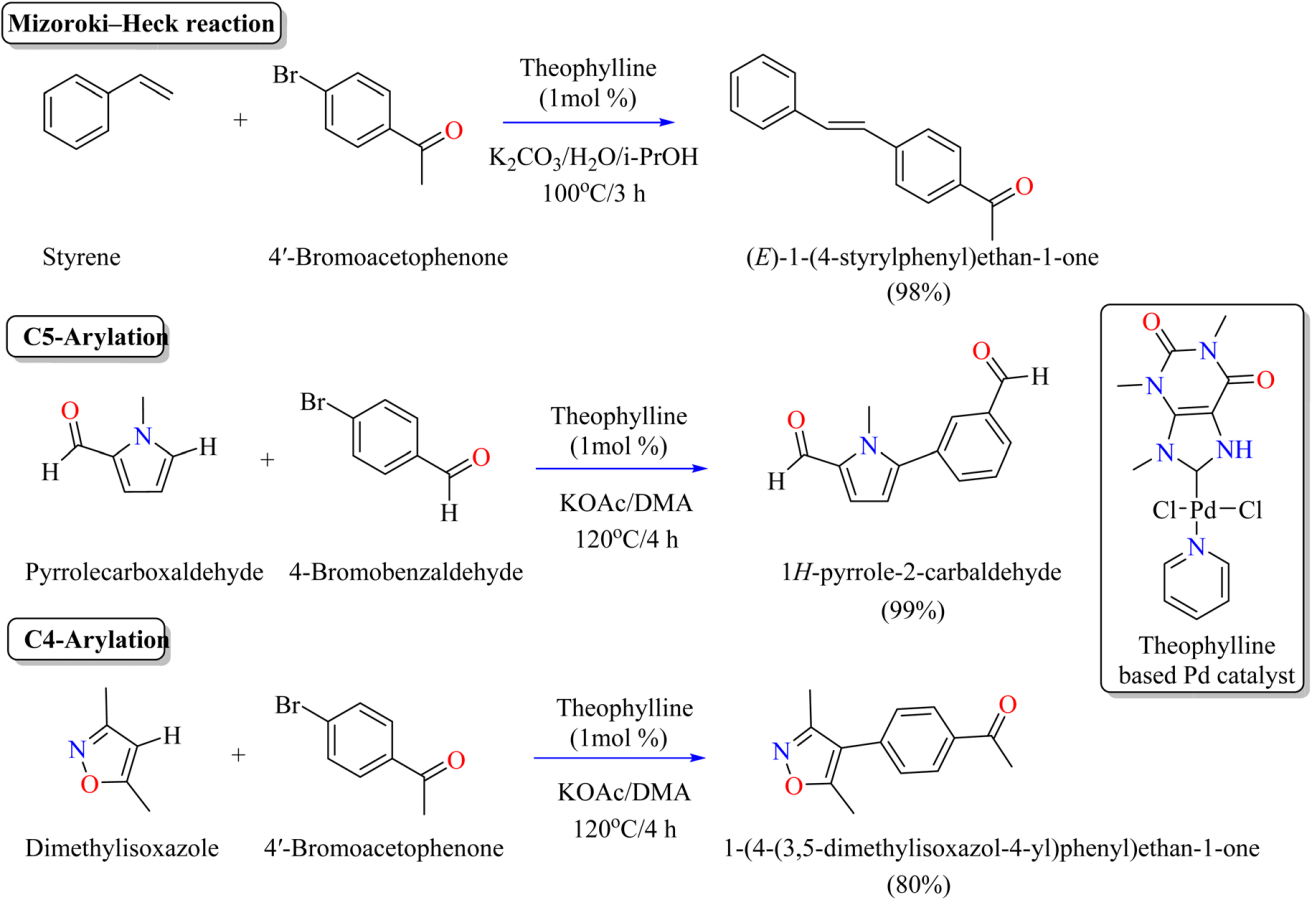
Theophylline-based Pd catalyzed synthesis of organic compounds.

Table 4 summarizes the theophylline-catalyzed synthesis of derivatives of organic compounds. Previously reported catalytic experiments showed that theophylline-based Pd complexes have strong catalytic activity in cross-coupling processes mediated by palladium, specifically the Suzuki–Miyaura coupling of aryl halides with phenylboronic acids.^107,108^ Researchers used mild, aerobic conditions and a low quantity of catalyst (0.25 mol%) in a green, water-based solvent with K_2_CO_3_ as a base to optimize these reactions.^109^ When these conditions were applied to aryl bromides, iodides, and chlorides bearing electron-withdrawing groups, the method efficiently yielded a variety of biaryl derivatives, though it required additional rigorous conditions. Scientists were motivated by these findings to investigate more difficult catalytic uses for promoting the Mizoroki–Heck reaction using this eco-friendly Pd–NHC complex. Researchers adjusted the cross-coupling of 4′-bromoacetophenone and styrene to assess the catalytic activity of theophylline in Mizoroki–Heck reactions. Microwave heating increased the conversion to 90%, even though the initial conversions were quite moderate. Standard parameters included 1 mol% catalyst, 100 °C, and 3 hours of microwave radiation (Scheme 5). Theophylline-based Pd catalyst was unable to successfully couple 4′-chloroacetophenone with styrene (1% conversion), since it merely promoted Suzuki–Miyaura coupling. This theophylline based catalyst was also used for the C5 and C4-arylation of pyrrolecarboxaldehyde and dimethylisoxazole, respectively, as shown in Scheme 5.^110^

**Table 4:** Summary of theophylline-based catalytic synthesis of organic compound derivatives

Synthesized compound	Reaction type	Reaction conditions	Reaction time	Product yield%
(*E*)-1-(4-Styrylphenyl)ethan-1-one	Mizoroki–Heck reactions	1 mol% of catalyst/K_2_CO_3_//100 °C	3 h	98
1*H*-Pyrrole-2-carbaldehyde	C5-Arylation	1 mol% of catalyst/KOAc/DMA/120 °C	4 h	99
1-(4-(3,5-Dimethylisoxazol-4-yl)phenyl)ethan-1-one	C4-Arylation	1 mol% of catalyst/KOAc/DMA/120 °C	4 h	80
Dioxodecahydroacridine	Hantzsch condensation reaction	15 mol% of catalysts/H_2_O/RT	10 min	98
1*H*-Pyrazolo[1,2-*b*]phthalazine-5,10-dione	One-pot reaction	Solvent free/70 °C	2 h	92
Pyrano-pyrimidinone	Knoevenagel–Michael cyclocondensation	15 mol% of catalyst, H_2_O/EtOH, 50 °C	10 min	88
Dihydropyrano-pyrazole	—	—	—	88
Tetrahydrobenzo-pyran	—	10 mol% of catalyst, H_2_O/EtOH, RT	—	89
Biphenyl	Suzuki–Miyaura coupling reaction	H_2_O/EtOH/NaCl 40 °C, air	30 min	Not mentioned
Tetrahydro-2′*H*-spiro[indoline-3,8′-pyrido[3,2-*d*]pyrimidine]-7′-carboxylate	Knoevenagel condensation	0.85 mol% of catalyst, H_2_O, reflux	8 h	95
Bis 3-amino-1*H*-benzo[*c*]pyrano[3,2-*a*]phenazine	—	0.035 g of catalyst, EtOH, reflux	30 min	94

**Scheme 6:**
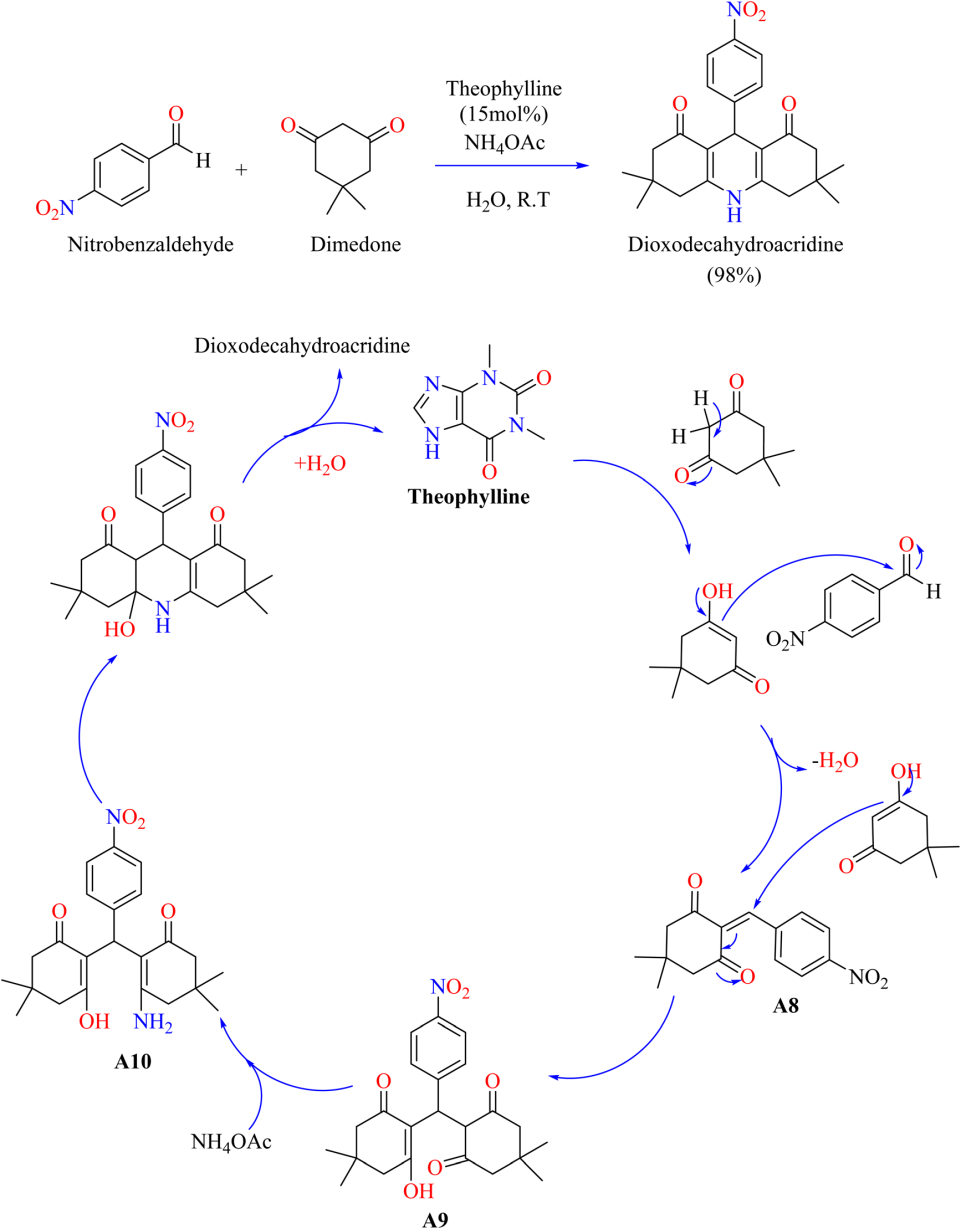
Methodology and reaction mechanism for the theophylline-catalyzed synthesis of dioxodecahydroacridine.

**Scheme 7:**
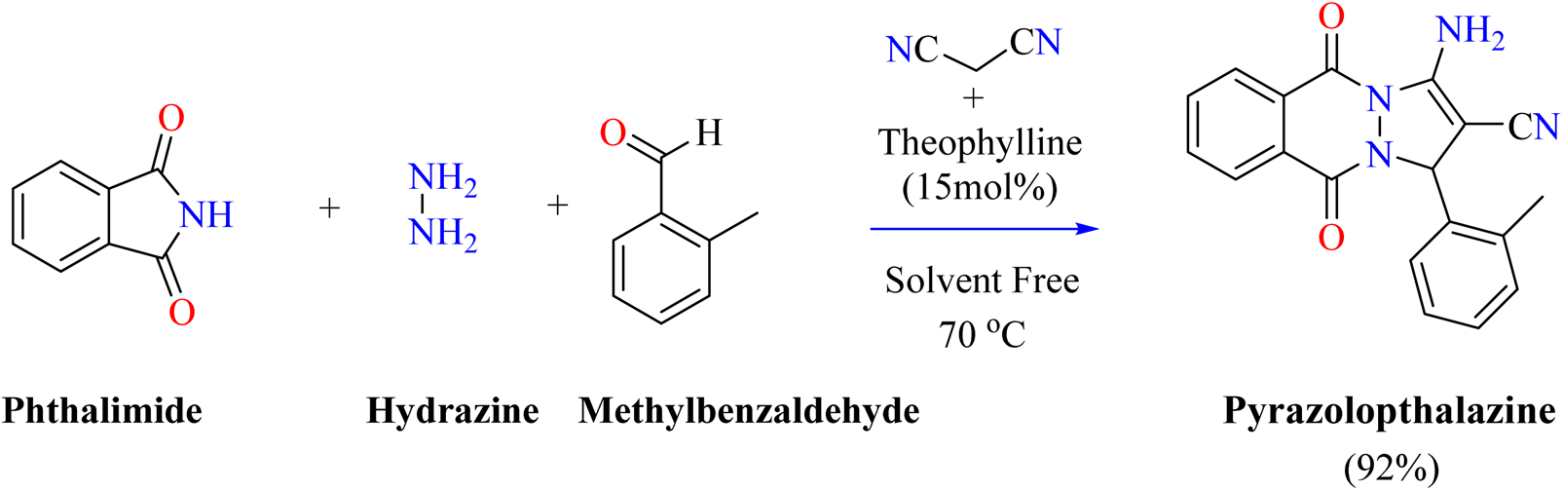
Synthesis of 1*H*-pyrazolo[1,2-*b*]phthalazine-5,10-dione.

Theophylline is a bio-based, environmentally friendly catalyst that provides a gentle and cost-effective way to synthesize 1,8-dioxodecahydroacridine through Hantzsch condensation. This environmentally friendly method has the advantages of being widely accessible, biodegradable, and simple to handle. This sustainable synthesis is improved by the methylxanthine drug theophylline, which treats respiratory disorders. It does this by offering an easy-to-use and gentle reaction environment for the synthesis of organic compounds.^111^ Researchers used theophylline as a solid base catalyst for achieving a more environmentally friendly synthesis of 1,8-dioxodecahydroacridine. Different solvents and catalyst concentrations were investigated in a three-component process involving ammonium acetate, dimedone, and *para*-nitrobenzaldehyde. Minimal products were produced when H_2_O was used without a catalyst. The investigated organic compound was synthesized efficiently in an aqueous medium at room temperature using 15 mol% theophylline as a catalyst, yielding optimal results. Comparing this method to the trace yield produced without the catalyst, there was a noticeable improvement. According to the reaction mechanism (Scheme 6), theophylline abstracts a proton from dimedone to facilitate the formation of intermediate (A8) through Knoevenagel condensation with *para*-nitrobenzaldehyde. To produce intermediate (A9), intermediate 8 was added to another dimedone by a Michael addition. Intermediate (A9) was then combined with ammonium acetate to produce intermediate (A10). The final product 1,8-dioxodecahydroacridine was finally produced *via* intramolecular cyclization.^112^ 1*H*-Pyrazolo[1,2-*b*]phthalazine-5,10-dione are useful heterocyclic compounds possessing a variety of biological actions, such as anti-inflammatory and anti-cancer effects.^113,114^ Catalysts such as NiCl_2_·6H_2_O and CuI nanoparticles have been utilized in a variety of ways for their synthesis; however, a number of these processes have disadvantages, including high costs and adverse environmental effects.^115–117^ Theophylline provides an economical, environmentally friendly, and biodegradable substitute. It catalyzes the solvent-free, four-component reaction in a single pot with good yields.^59,111,118^ As shown in Scheme 7, Farzaneh heated theophylline (15 mol%), hydrazine monohydrate, and phthalimide for two hours at 70 °C. Then, malononitrile and aromatic aldehyde were added, and the reaction was heated until it was finished, as seen by TLC. To achieve a pure compound, the resulting mixture was cooled, filtered, and the products were recrystallized from ethanol.^119^

**Scheme 8:**
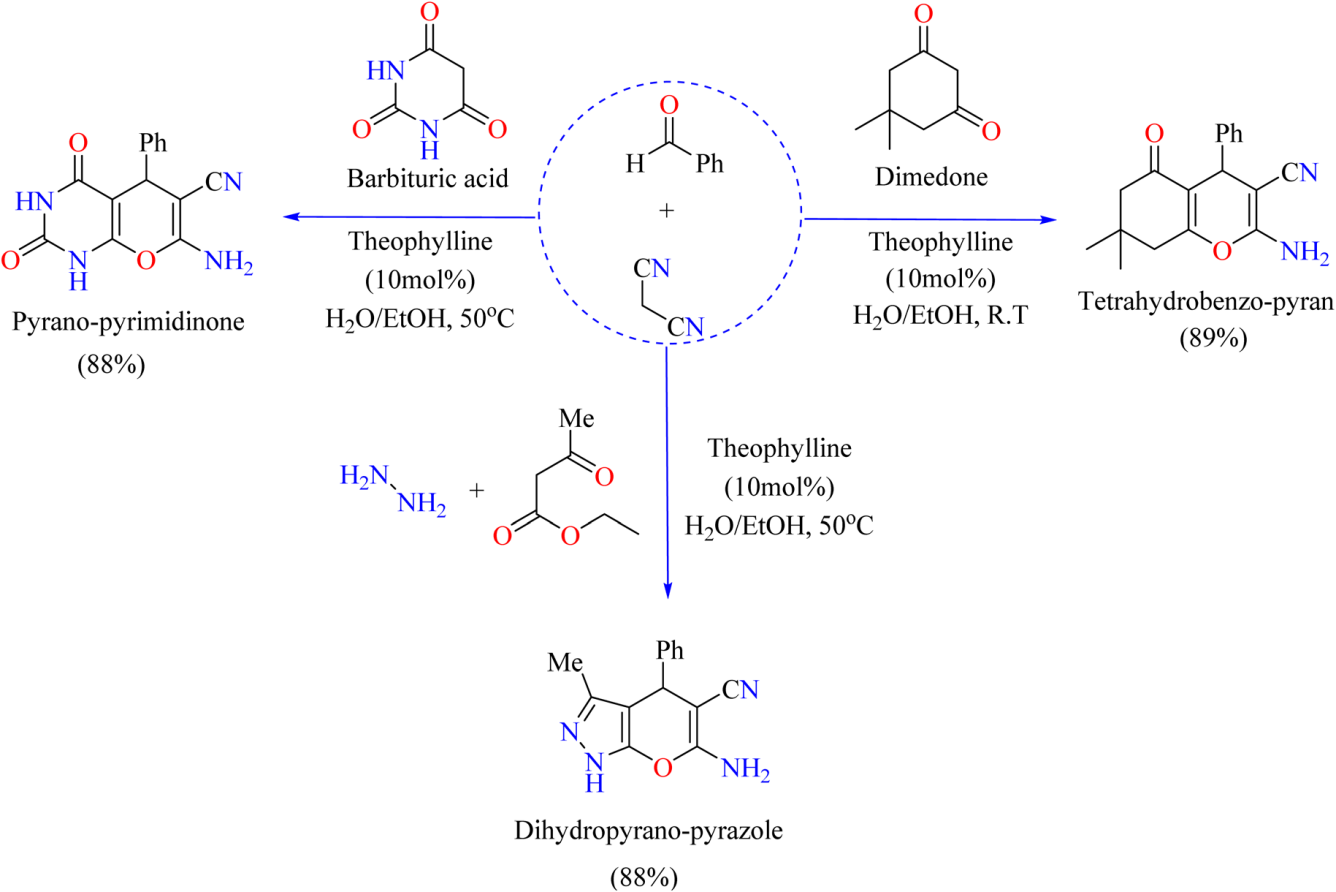
Methodology for the synthesis of theophylline-catalyzed pyran annulated heterocyclic compounds.

Pyran derivatives are prized for their wide range of medicinal properties, which include anti-inflammatory and anti-cancer properties.^120,121^ Several techniques utilizing diverse catalysts such as ZnO NPs, SiO_2_ NPs, and CaHPO_4_ have been documented for their synthesis.^122–125^ Nevertheless, low yields, protracted reaction times, unfavorable environmental conditions, and excessive reagent use are common problems with these techniques. Thus, it is imperative to create synthesis techniques that are milder, more ecological, and more effective. Using theophylline as a green catalyst, scientists have synthesized an environmentally friendly process that produces good yields of pyran-annulated heterocyclic compounds in aqueous/EtOH media, as shown in Scheme 8. In the case of pyrano-pyrimidinone synthesis, theophylline (15 mol%) was reacted with benzaldehyde, barbituric acid, and malononitrile in aqueous ethanol at 50 °C. The products were filtered, washed, and recrystallized. Dihydropyranopyrazole was synthesized by the reaction of benzaldehyde, malononitrile, hydrazine hydrate, and ethyl acetoacetate in H_2_O/EtOH at 50 °C, and was catalyzed by theophylline (15 mol%). The product was cooled before being filtered, cleaned, and recrystallized. To synthesize tetrahydrobenzo[*b*]pyran, theophylline (10 mol%) was used as a catalyst at room temperature in a reaction with dimedone, malononitrile, and benzaldehyde, following the previously described procedure.^126^

**Scheme 9:**
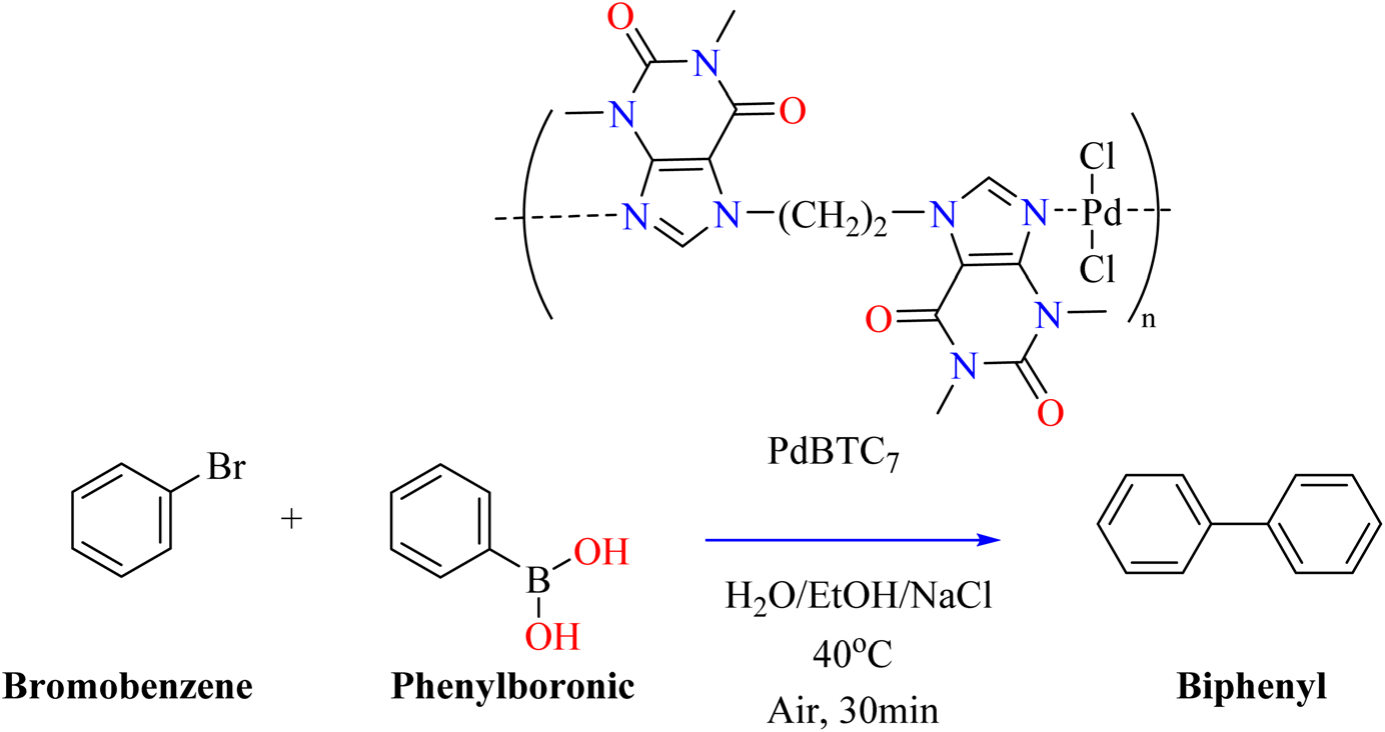
Bistheophylline catalyzed synthesis of biphenyl.

Metal–N bonding allows purine derivatives like theophylline and caffeine to form stable metal complexes with transition metals.^127^ These substances can coordinate with palladium, just like N-heterocyclic carbenes (NHCs); however, in these complexes, palladium forms a bond with the imidazole ring's carbon.^128^ Recent research demonstrates that theophylline may accelerate Suzuki–Miyaura C–C coupling reactions even in acidic environments by forming very stable Pd(ii) complexes *via* Pd–N chelation.^129,130^ Lately, (heptane-1,7-diyl)bistheophylline (PdBTC_7_) and palladium dichloride complex have been found to produce a stable complex that forms micro-sized flower-like structures in aqueous mediums. PdBTC_7_ is stable in air and insoluble in typical organic solvents and acidic environments.^131^ Bistheophylline has great catalytic activity and extraordinary recyclability as a heterogeneous catalyst for Suzuki–Miyaura C–C coupling processes. This is especially noticeable when NaCl is added, which reduces Pd leaching and improves performance by stabilizing the Pd complex.^132^ The research investigated a circulation reactor system with glass bead-packed bistheophylline microflowers arranged in a column. NaCl improved the performance and stability of this setup, which effectively catalyzed the coupling of bromobenzene and phenylboronic acid. Biphenyl product was constantly produced by injecting 51 injections of phenylboronic acid and bromobenzene into an eluent consisting of water, NaCl, and alcohol (Scheme 9). Excellent performance and stability were demonstrated by the catalyst, which showed a turnover number (TON) of 4498 and a turnover frequency (TOF) of 8996 h^−1^.^133^

**Scheme 10:**
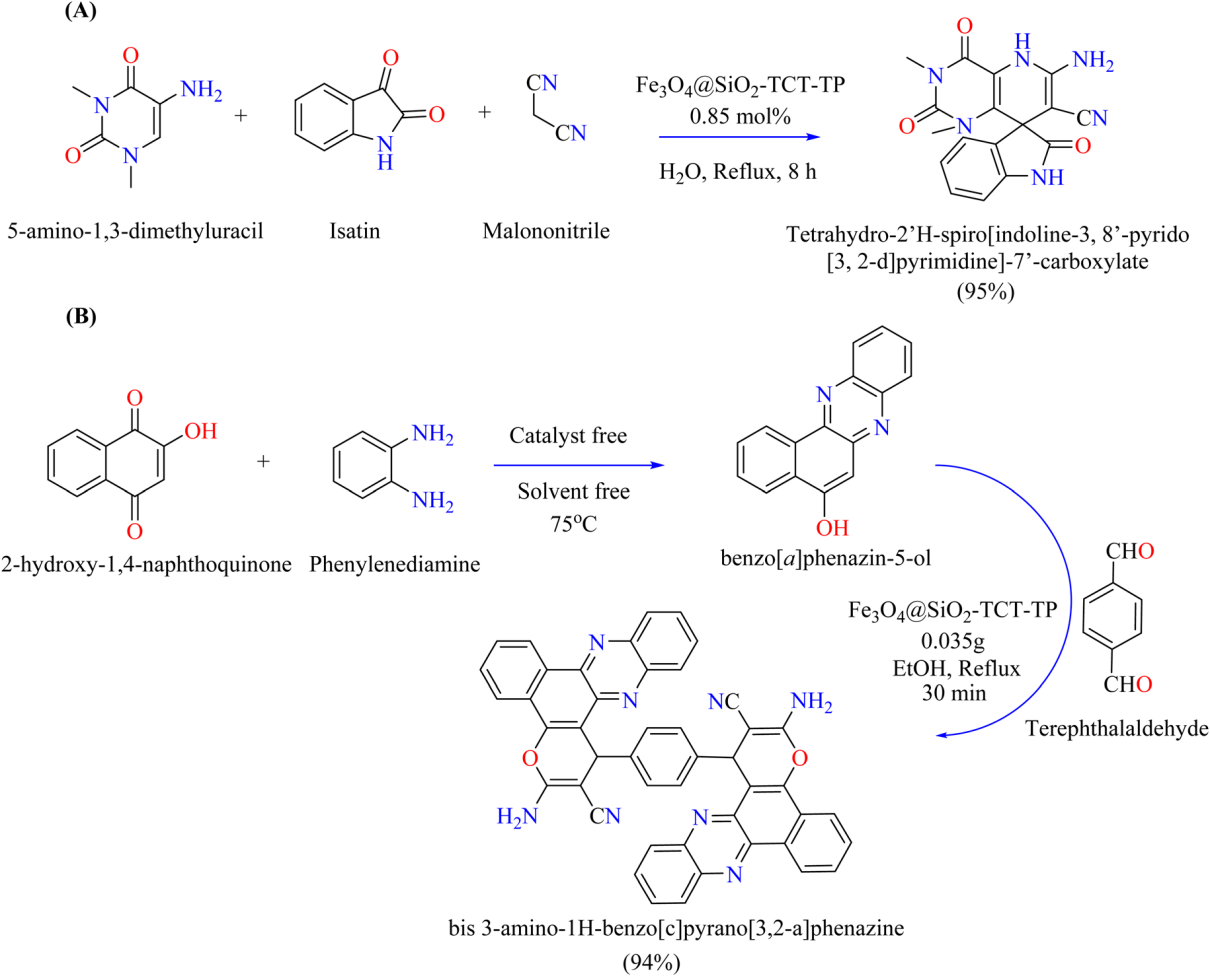
Theophylline NPs catalyzed the synthesis of organic compounds. (A) Synthesis of tetrahydro-2′*H*-spirooxindoles-3,8-pyrido-[3,2-*d*]pyrimidine]-7′-carboxylate. (B) Synthesis of bis 3-amino-1*H*-benzo[*c*]pyrano[3,2-*a*]phenazine.

Important heterocyclic compounds, phenazines and pyrans have a wide range of biological activity and uses, such as in natural goods, drugs, and colors.^134–136^ While pyrans are recognized for their anticoagulant, antioxidant, and central nervous system activity, among other things, phenazines show antibacterial, anticancer, and anti-inflammatory qualities.^137–140^ Few compounds incorporated both the pyran and phenazine structures, despite their significance. Theophylline nanoparticles (NPs) were evaluated as a magnetic heterogeneous catalyst for the production of spirooxindoles in one pot using three components. The conditions were tuned under reflux using 5-amino-1,3-dimethyluracil, isatin, and malononitrile as a model reaction. Reactions were tested using different isatin derivatives and activated methylene compounds such as ethyl cyanoacetate to assess the scope of the substrate and the adaptability of the approach. A boiling water with 1 mmol of each component was the optimal condition for 0.85 mol% Fe_3_O_4_@SiO_2_–TCT–theophylline catalyst (Scheme 10A). Conversely, 2-hydroxy-1,4-naphthoquinone, *o*-phenylenediamine, malononitrile, and terephthalaldehyde were reacted in ethanol (8 mL) with Fe_3_O_4_@SiO_2_–TCT–theophylline (0.035 g) under reflux conditions to synthesize bis 3-amino-1*H*-benzo[*c*]pyrano[3,2-*a*]phenazine, as shown in Scheme 10B.^141^

## 3 Neurological effects

Theophylline's impact on neurotransmitter systems and possible therapeutic uses have made it a promising drug in neurological contexts. Theophylline stimulates dopamine signaling, an adenosine receptor antagonist that is essential for cognitive and motor function.^142^ Because dopamine imbalance is a characteristic of neurological illnesses like Parkinson's disease, this method is very helpful in treating these conditions. Adenosine's function in the brain is also modulated by theophylline, which may help treat Alzheimer's disease by enhancing neural plasticity and lowering neurological inflammation.^143,144^ Moreover, theophylline raises cyclic AMP (cAMP) levels *via* inhibiting phosphodiesterase (PDE) enzymes. Since this increase in cAMP is associated with better cognitive function, theophylline may be used as a treatment for neurodegenerative illnesses and cognitive decline.^145,146^ Theophylline's neuroprotective qualities and capacity to improve memory and learning through PDE inhibition lend credence to its potential as a treatment for diseases, including dementia and age-related cognitive impairment.^147^ Theophylline's neurobiological effects imply that it may be useful in treating neurological illnesses by altering important brain pathways related to neurotransmission and cognitive function, in addition to its use as a bronchodilator.

Beyond this, the wide range of side effects that are usually associated with plasma concentrations higher than 20 mg L^−1^ makes theophylline use commonly restricted.^148^ However, some people may still have adverse consequences even at lower plasma levels. To achieve therapeutic concentration with the least amount of side effects, a progressive dose escalation is advised. Effectiveness and tolerability in individuals receiving theophylline are balanced with the aid of this strategy. Theophylline frequently causes headache, nausea, vomiting, upset stomach, and agitation as adverse effects.^149,150^ Increased acid secretion, acid reflux, and diuresis are possible side effects. It can result in cardiac rhythms and convulsions at high concentrations.^151^ Furthermore, there are worries that the infusion of intravenous aminophylline in emergencies could increase the risk of mortality in cases of severe asthma.^152^ Theophylline is mostly used to treat respiratory disorders. Because it particularly blocks A1A receptors, its adenosine receptor antagonism can lead to adverse effects such as diuresis, increased gastric output, central stimulation, and arrhythmias. Doxofylline or PDE inhibitors can reduce these effects by focusing on distinct pathways. Theophylline's most prevalent adverse effects include headaches and nausea, which are related to its inhibition of several phosphodiesterases (PDEs), such as PDE3, which alters heart rhythm, and PDE4, which is located in the vomiting center. PDE inhibitors and other alternative therapies may help lower theophylline's side effects because of the drug's intricate interactions with phosphodiesterases and adenosine receptors.^153^ Theophylline's industrial and therapeutic potential should be maximized by future research that focuses on improving its pharmacokinetics, decreasing dose-related adverse effects, and increasing its stability in catalytic applications.

## 4 Conclusions and outlook

Theophylline's mild reaction conditions, efficiency, and selectivity make it an advantageous biobased catalyst in synthetic and medicinal chemistry. They follow the “green chemistry” criteria of being economical, eco-friendly, and produced from renewable resources utilizing supercritical CO_2_ and water. Theophylline's biological functions, which may include antibacterial and anticancer effects, add to its significance in environmental and medicinal contexts beyond its catalytic uses. Synthetic complexes based on theophylline exhibit superior potential as antibacterial and anticancer agents in contrast with traditional medicines and theophylline alone. In addition to increasing its usefulness in intricate chemical processes, theophylline effectively catalyzes multi-component reactions in one-pot synthesis. Its advantages in synthetic and medicinal chemistry applications are highlighted by this dual capability. Theophylline, which was once less popular in developed countries, may see a renewed interest in treatment for severe asthma, smoking asthma, and COPD due to its possible anti-inflammatory and immunomodulatory benefits at low dosages (5–10 mg L^−1^).^154,155^ It is less problematic with drug interactions, easier to take, and has fewer adverse effects at these levels. In individuals with severe asthma, smoking asthma, and COPD where corticosteroids are less effective, they may operate in concert with corticosteroids due to their anti-inflammatory properties by restoring HDAC activity.^156^ Furthermore, leukotriene modifiers and long-acting inhaled β2-agonists are more expensive than slow-release theophylline, and oral medication may improve compliance. Therefore, low-dose theophylline may be a useful supplementary therapy for many diseases.^157^ Theophylline is anticipated to be investigated as a catalyst for reactions involving a range of substrates in light of recent developments. New types of catalysts and antibacterial agents based on theophylline may also be developed.

## Ethical statement

The researchers sought informed consent from all participants before recruitment for data collection.

## Consent for publication

The explicit consent for publication was obtained from participants.

## Data availability

No primary research results, software, or code has been included and no new data were generated or analyzed as part of this review.

## Author contributions

Abdul Ahad: writing – original draft. Adnan Majeed: writing review & editing, software, and data curation. Ayesha Zafar: writing – reviewing, validation, and software. Muhammad Adnan Iqbal: conceptualization, resources, and supervision. Shahzaib Ali: formal analysis. Muneeba Batool: visualization. Asma Rehman: data curation. Faiza Manzoor: formal analysis.

## Conflicts of interest

The authors declare that they have no competing interests.
